# Advances in immune checkpoint inhibitors therapy for small cell lung cancer

**DOI:** 10.1002/cam4.5659

**Published:** 2023-03-07

**Authors:** Longhui Li, Yangyueying Liang, Minghui Yu, Lu Zhao, Qingyun Mei, Yongchao Yu, Na Wang, Dou Zhang, Ziwei Wang, Yingjie Jia, Fanming Kong

**Affiliations:** ^1^ Department of Oncology First Teaching Hospital of Tianjin University of Traditional Chinese Medicine Tianjin China; ^2^ National Clinical Research Center for Chinese Medicine Acupuncture and Moxibustion Tianjin China

**Keywords:** clinical trials, immune checkpoint inhibitors, SCLC, tumor microenvironment

## Abstract

**Background:**

As one of the most aggressive neuroendocrine tumors, small cell lung cancer (SCLC) has the most disappointing prognosis of all lung cancers. Although SCLC responds well to initial chemotherapy, the majority of patients experience disease recurrence within one year, and patient survival is poor. It is still necessary to explore the application of ICIs in SCLC since the beginning of the road to immunotherapy, which broke the 30‐year treatment deadlock of SCLC.

**Methods:**

We searched PubMed, Web of Science, and Embase with search terms such as "SCLC", "ES‐SCLC", "ICIs", and "ICBs", and categorized and summarized the relevant literature obtained, and we compiled the latest progress about the application of ICIs in SCLC.

**Results:**

We listed 14 clinical trials on ICIs, including 8 clinical trials on first‐line SCLC treatment, 2 clinical trials on second‐line SCLC treatment, 3 clinical trials on third‐line SCLC treatment, and 1 clinical trial on SCLC maintenance treatment.

**Conclusion:**

ICIs in combination with chemotherapy can improve OS in SCLC patients, but the extent to which SCLC patients can benefit from ICIs is limited, and ICIs' combination treatment strategies still need to be continuously explored.

## Graphical abstract

In recent years, research on immune checkpoint inhibitors (ICIs) for small cell lung cancer (SCLC) has never stopped, experimental drugs and clinical trials have increased year after year, and treatment modalities have gradually diversified. This review summarized the current state of ICIs for SCLC and prospected for the future treatment direction.

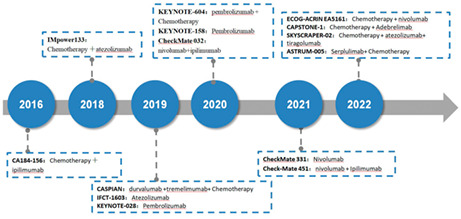

## 1 INTRODUCTION

According to the latest National Cancer Center of China data, lung cancer is the most frequent tumor in China with a high incidence and fatality rate.^1^ Small cell lung cancer (SCLC) was one of the most dangerous subtypes of lung cancer, accounting for about 15%–20% of all cases.^2^ Tobacco use was strongly connected to the development of SCLC, and there is evidence that the mutational nature of SCLC has a distinct smoking profile.^3^ Almost all patients have a smoking history, and only 2% of SCLC patients do not smoke.^4^ SCLC increases more rapidly, is more aggressive, has earlier metastasis, and responds better to radiotherapy, but has a short remission period and a high recurrence rate. SCLC can be divided into four main distinct molecular subtypes.^5^ Approximately 70% of all SCLC patients were in the extensive stage, and the mOS for extensive‐stage SCLC(ES‐SCLC) is significantly shorter than that for limited‐stage SCLC (LS‐SCLC).^6^ The overall prognosis of ES‐SCLC was not promising, with a 2‐year survival rate of approximately 2%.^2^

Cytotoxic T lymphocyte‐associated antigen 4(CTLA‐4), programmed cell death protein 1(PD‐1), and programmed cell death ligand 1(PD‐L1) inhibitors have received a lot of attention and application in recent years.^7^ PD‐1 (CD279) and PD‐L1 (CD274 and B7‐H1) were immune response suppressors that, when combined, could suppress tumor immunity and induce T‐cell apoptosis via a variety of mechanisms. Blocking CTLA‐4, a negative regulator enhances the proliferation and maintenance of CD8^+^ T cells.^8^, ^9^ Combining PD‐1/PD‐L1 inhibitors can stop negative regulatory signaling, restoring T cells' ability to fight tumors. TIGIT is a promising novel immunotherapy target for cancers. The infiltration of tumor‐infiltrating lymphocytes (TILs) was correlated with the clinical impact of immune checkpoint inhibitors(ICIs).^5^ TIGIT, which was co‐expressed on TILs with PD‐1, could suppress immunity and promote tumor growth via multiple mechanisms. The simultaneous use of PD‐1 and TIGIT now appears to be very promising because TIGIT inhibition or in combination with PD‐1 inhibition primarily works on NK cells to promote antitumor response and inhibit cancer progression.^10^

ICIs provided hope for the treatment of SCLC, and a growing body of clinical evidence suggested that ICIs could improve SCLC patients' prognoses. We present the background of TME, review some clinical trials of ICIs for SCLC, and provide an outlook on future treatment modalities for SCLC in this article. It is expected to make recommendations for the treatment of SCLC.

### 1.1 The tumor microenvironment in SCLC

The tumor microenvironment (TME) was a dynamic and complex ecosystem that plays a role in tumorigenesis and metastasis.^11^ Innate immune cells, acquired immune cells, and tumor cells were the main constituents.^12^, ^13^ Immune cells were crucial in the tumorigenesis process. Tumor‐associated immune cells were involved in both inhibiting tumor growth and promoting tumor progression. Tumor immune cells destroy cancer cells early in tumorigenesis, but cancer cells could promote tumorigenesis by suppressing immune cells' cytotoxic effects.^14^

SCLC was characterized by a large degree of intra‐tumor heterogeneity, with high expression of regulatory T cells (Tregs) and low expression of MHC class I molecules as distinctive features.^12^, ^15^, ^16^ Poor differentiation of the vasculature in SCLC, inadequate nutrient supply, and inability to remove metabolic waste promptly caused an adverse TME.^11^ SCLC had obvious immunosuppression and multiple immune cells, such as CD45^+^ T cells, Tregs, Cytotoxic T lymphocytes (CTLs), and TILs when compared to other types of lung cancer.^17^ Typically, the expression of the transcription factor Forkhead box protein P3 (FoxP3) served to identify Tregs. Tregs were a subpopulation of CD4^+^ T cells. Only the continuous expression of FoxP3 could exert the ability to label Tregs and control Tregs' capacity.^18^, ^19^ SCLC cells could produce IL‐15, which could activate Tregs while inhibiting CD4^+^ T cell growth.^20^ Tregs in general could contribute to immune dynamic homeostasis. However, Tregs could express CTLA‐4 on their surface in TME, and CTLA‐4, when bound to B7 on the surface of antigen‐presenting cells (APCs), could inhibit further activation of T cells. Tregs were also able to express PD‐L1, which, when combined with PD‐1 expressed on the surface of APCs and T cells, could activate the immune checkpoint pathway, cause tumor immune escape, and promoted tumor progression.^16^, ^18^, ^21^, ^22^ CTLA‐4, PD‐1, and PD‐L1 inhibitors could block such effects of Tregs to achieve anti‐tumor.^18^, ^23^

Lymphocytes that had infiltrated into tumors expressing PD‐L1 were found to be positively correlated with relapse‐free survival in a study of protein expression in surgically removed tissues from patients with SCLC. Patients with positive PD‐L1 expression also had a significantly better long‐term prognosis than those with negative PD‐L1 expression and a low proportion of lymphocytes.^24^ Very little PD‐L1 was expressed by SCLC tumor cells, and its usefulness as a biomarker for predicting ICIs response was debatable. The ICIs response was associated with high PD‐L1 expression in the NCT02628067 and NCT02359019 trials, but this was not confirmed in the NCT02763579, NCT03066778, NCT03043872, and NCT01928394 trials.^2^ There was an argument over the clinical significance of PD‐L1 expression, which was higher in immune or mesenchymal cells than in tumor cells.^2^, ^22^, ^25^ There was limited data on TME, and differences in TME cause ICIs to be used less widely in SCLC than in Non‐SCLC (NSCLC). Further investigation of TME, followed by the identification and refinement of biomarkers, is required before precision treatment of SCLC can be realized.^26^

In the future, TME can be used to monitor efficacy and predict secondary resistance, and to continually investigate the mechanisms of ICIs resistance and develop new drugs. A finer stratification of SCLC patients will be performed based on various factors such as age, TNM stage, TME characteristics, and molecular subtypes of SCLC to determine the best beneficiary population for ICIs.

### 1.2 First‐line treatment

Ipilimumab was the first drug to be tried in first‐line immunotherapy in ES‐SCLC. A phase III clinical trial found that adding ipilimumab to chemotherapy did not improve overall survival (OS). The trial randomized 1132 patients proportionally to receive chemotherapy + ipilimumab or chemotherapy + placebo. For chemotherapy plus ipilimumab versus chemotherapy plus placebo, the median overall survival (mOS) was 11 and 10.9 months, respectively (HR = 0.94, *p* = 0.3775). The A arm had higher rates of treatment‐related discontinuation; 82% and 76% of patients, respectively, experienced adverse events (AEs) from their medications. In the A arm, there were more grade 3–4 cases than in the Control arm (48% vs. 44%). The study also did not show significant improvements in other endpoints.^27^

In the IMpower‐133 study, 403 patients were randomly assigned to receive chemotherapy plus Atezolizumab or chemotherapy plus placebo, with OS and Progression‐free survival(PFS) as the primary endpoints. The median PFS(mPFS) was 5.2 months and 4.3 months in the two groups, respectively (HR = 0.77, *p* = 0.02), and the mOS was 12.3 months and 10.3 months, respectively, in the A arm versus the Control arm (HR = 0.70, *p* = 0.007).^28^ Comparing patients in the Control arm to those in the A arm, the 1‐year survival rate increased from 38.2% to 51.7%, and the quality of life for those patients was better. Atezolizumab's associated toxicity and symptom burden were not found to have increased in the updated study results.^29^

In the CASPIAN study, 805 untreated ES‐SCLC patients were proportionally divided into arms receiving chemotherapy plus Durvalumab and Tremelimumab, chemotherapy plus Durvalumab, or chemotherapy alone.^30^ The results of this trial showed that the mOS was 2.7 months higher in the B arm than in the Control arm, the OS rates at 12 and 18 months were also higher than in the Control arm, and the overall safety was similar in both groups.^30^ On March 30, 2020, the FDA formally approved Durvalumab in conjunction with chemotherapy for the treatment of ES‐SCLC. According to the updated result, there was no discernible distinction in overall survival rates between the A arm and the Control arm (HR = 0.82, *p* = 0.045), and common neutropenia was more common in the A arm than in the B arm (32% vs. 24%).^31^ According to a recent analysis of CASPIAN, B, A, and Control arms had median OS of 12.9 months, 10.4 months, and 10.5 months, respectively, with PFS of 5.1 months, 4.9 months, and 5.4 months, Patients in the B arm achieved a 36‐month OS rate of 15.3 percent and a 3‐fold survival rate at 3 years compared to the Control arm, to solidify Durvalumab in combination with chemotherapy as the first‐line treatment standard for ES‐SCLC.^32^

The primary endpoints of the KEYNOTE‐604 study were PFS and OS. The clinical trial involved 453 patients, with 228 randomly assigned to the PD‐1 inhibitor Pembrolizumab + chemotherapy arm and 225 assigned to the placebo+chemotherapy arm. The mPFS in the A arm was 4.5 months and 4.3 months in the Control arm, respectively (HR = 0.75, *p* = 0.0023). The 12‐month and 24‐month OS rates in the A arm were 45.1% and 22.5%, respectively, compared to 39.6% and 11.2% in the Control arm, but the mOS in the A arm versus the Control arm was 10.8 and 9.7 months, respectively (HR = 0.80, *p* = 0.0164), which fell short of the expected *p* = 0.0128.^33^

The ECOG‐ACRIN EA5161 clinical trial involved 160 patients with primary SCLC and an ECOG functional level of 0 or 1. PFS was the study's primary endpoint. The mPFS of the A arm and the Control arm was 5.5 months and 4.7 months (HR = 0.68, *p* = 0.047), respectively, and the mOS was 11.3 months and 8.5 months (HR = 0.67, *p* = 0.038). Results were similar to those of KEYNOTE‐604 only with improved PFS and no statistically significant OS difference.^34^

The Capstone‐1 study^35^ was a clinical trial with the primary study endpoint of OS, which was conducted in 47 hospitals in China. A total of 462 patients with untreated SCLC with ECOG functional level 0 or 1 were included, with 230 patients randomly assigned to receive Adebrelimab (SHR‐1316) in conjunction with chemotherapy, and 232 patients receiving placebo + chemotherapy. The findings of the study showed that the mOS in the A arm was significantly longer than that in the Control arm (15.3 vs. 12.8 months), and the mPFS was 5.8 months and 5.6 months respectively. With OS rates of 62.9% and 31.3% in the A arm and 52.0 percent and 17.2 percent in the Control arm at 12 and 24 months, respectively, and PFS rates of 49.4% in the A arm and 37.3% in the Control arm at 6 months and 19.7% and 5.9%, respectively. At 12 months, Adebrelimab combined with chemotherapy was able to significantly improve the OS rate of patients, extending their OS by 2.5 months.

A total of 490 patients were enrolled in the SKYSCRAPER‐02 study, and they were randomly assigned to the Atezolizumab + Tiragolumab + chemotherapy arm or the Atezolizumab + chemotherapy arm. As of February 6, 2022, the study's findings showed no benefit in mPFS and OS in untreated ES‐SCLC patients, and the addition of Tiragolumab did not yield any additional benefit.^36^ In comparison to the SKYSCRAPER‐02 study, the IMpower133 study did not include patients with brain metastases. We can conclude from the results of the SKYSCRAPER‐02 study that Atezolizumab in combination with EP remains the standard for first‐line therapy and that Atezolizumab + chemotherapy + Tigit inhibitor (Tiragolumab) did not add any additional OS or PFS benefit. It also compensated for the fact that the IMPower133 study excluded individuals suffering from brain metastases. Another phase III trial comparing Tigit inhibitors, KeyVibe‐008 (NCT05224141), is currently underway.^37^

The ASTRUM‐005 was a clinical trial with the primary endpoint of OS. And 585 patients with untreated ES‐SCLC were randomly assigned to receive either Serplulimab, a PD‐1 inhibitor, in combination with chemotherapy, or a placebo, in a 2:1 ratio. The mOS in the Serplulimab + chemotherapy arm was 15.4 months and 10.9 months in the placebo + chemotherapy arm, respectively (HR = 0.63, *p* = 0.001). OS rates were 60.7 percent and 43.1 percent, respectively, at 12 and 24 months in the Serplulimab + chemotherapy arm, and 47.8% and 7.9%, respectively, in the placebo + chemotherapy group. The mPFS was 5.7 months and 4.3 months, respectively, in the two groups (HR = 0.48; *p* = 0.001). There was no appreciable rise in serious AEs, which is similar to other PD‐1 and PD‐L1 monoclonal antibodies in terms of safety. Serplulimab reached a new record for OS in first‐line therapy of ES‐SCLC, with a two‐year OS rate that exceeded that of the chemotherapy arm by a factor of five, making it the first PD‐1 monoclonal antibody to generate good results in ES‐SCLC. As a result of this finding, the FDA designated Serplulimab as an orphan medication for ES‐SCLC.^38^, ^39^

When compared to typical chemotherapy regimens, which only extended the OS by 2 months, the phase III clinical outcomes of IMpower133 and CASPIAN revealed comparable mOS of 12.3 and 12.9 months, respectively^28^, ^30^, ^32^ (Table 1). This does not meet our desire for a longer OS. The mOS in the Serplulimab paired with the chemotherapy group in the ASTRUM‐005 study was 15.4 months, which was 4.5 months longer than in the placebo group.^39^ The two‐year OS rate of 43% was even more amazing. In the control group, this figure was only 7.9%.^39^ The ASTRUM‐005 study currently has the longest OS in the first‐line treatment of ES‐SCLC.

**TABLE 1:** First‐line treatment for SCLC

NCT ID	Stage	Number	Never Smoking Treatment arm (percent)	Treatment	Chemotherapy cycle	Endpoint	Progression‐Free‐Survival (month)	overall survival (month)
NCT01450761 (CA184‐156)	III	1132	36	A: Chemotherapy + ipilimumab; Control: Chemotherapy +placebo	4	OS	A:4.6; Control: 4.4; Hazard ratio 0.85, *p* = 0.0161	A:11; Control:10.9; Hazard ratio 0.94, *p* = 0.3775
NCT02763579 (IMpower133)	III	403	4.5	A: Chemotherapy +atezolizumab; Control: Chemotherapy + placebo	4	PFS, OS	A:5.2; Control: 4.3; Hazard ratio 0.77, *p* = 0.02	A:12.3; Control: 10.3; Hazard ratio 0.70, *p* = 0.007
NCT03043872 (CASPIAN)	III	805	A:6; B:8	A: durvalumab + tremelimumab+ Chemotherapy; B: durvalumab+ Chemotherapy; Control: Chemotherapy	A:4; B:4; Control: up to 6	OS	A:4.9; B:5.1; Control:5.4; A VS control: HR, 084 *p* not tested; B VS control: HR 0.78; *p* not tested	A:10.4; B:12.9; Control: 10.5; A VS Control:; HR 0.82, *p* = 0.045; B VS Control:; HR 0.75, *p* = 0.0032
NCT03066778 (KEYNOTE‐604)	III	453	3.5	A: pembrolizumab + Chemotherapy; Control: Chemotherapy + placebo	4	PFS, OS	A:4.5; Control: 4.3; Hazard ratio 0.75, *p* = 0.0023	A:10.8; Control: 9.7; Hazard ratio 0.80; *p* = 0.0164
NCT03382561 (ECOG‐ACRIN‐EA5161)	II	160	NA	A: Chemotherapy + nivolumab; Control: Chemotherapy	4	PFS	A:5.5; Control: 4.7; Hazard ratio 0.68, *p* = 0.047	A:11.3; Control: 8.5; Hazard ratio 0.67, *p* = 0.038
NCT03711305 (CAPSTONE‐1)	III	462	22	A: Chemotherapy + Adebrelimab; Control: Chemotherapy + placebo	4–6	OS	A:5.8; Control: 5.6; Hazard ratio 0.67, *p* < 0.0001	A:15.3; Control:12.8; Hazard ratio 0.72; *p* = 0.0017
NCT04256421 (SKYSCRAPER‐02)	III	490	NA	A: Chemotherapy + atezolizumab+ tiragolumab Control: Chemotherapy + atezolizumab+placebo	4	PFS, OS	A:5.4; Control: 5.6; Hazard ratio 1.11, *p* = 0.3504	A:13.6; Control: 13.6; Hazard ratio 1.04; *p* = 0.7963
NCT04063163 (ASTRUM‐005)	III	585	20.8	A: Serplulimab+ Chemotherapy; Control: placebo+ Chemotherapy	up to 4	OS	A:5.7; Control: 4.3; Hazard ratio 0.48, *p* < 0.0001	A:15.4; Control:10.9; Hazard ratio 0.63; *p* < 0.001

Serplulimab, a Chinese‐made intravenous PD‐1 monoclonal antibody, was the first PD‐1 monoclonal antibody to demonstrate efficacy and positive results in the treatment of SCLC.^40^ Serplulimab, which received a license in China in March 2022, was similar to other PD‐1 monoclonal antibodies in the binding region of PD‐L1, preventing PD‐1 from interacting with ligands PD‐L1 and PD‐L2, and offering dose‐dependent tumor suppression.^40^, ^41^ The survival curves of the Durvalumab and Atezolizumab combination chemotherapy groups started to separate from the control group after 6 months of treatment, whereas the Serplulimab combination chemotherapy group demonstrated a significant separation after 4 months of treatment, and the difference between the two groups was significant and remained at a higher position.^38^ Not only did the OS curves achieve excellent results, but the results of the PFS curves were also striking, with separation and crossover in the PFS curves of the Durvalumab and Atezolizumab combination chemotherapy groups after 4 months of treatment. In contrast, at 2 months of treatment, the PFS curves in the Serplulimab combination chemotherapy group were separated and uncrossed. There was no substantial increase in serious AEs, as with Durvalumab and Atezolizumab^38^ (Table 2).

**TABLE 2:** Differences between IMpower133, CASPIAN, and ASTRUM‐005.

NCT ID	Treatment	Never Smoking Treatment arm (percent)	Treatment‐related adverse events (grade ≥3, percent)	Immune‐related adverse events (grade ≥3, percent)	Overall survival rate (percent)	OS benefit (month)
NCT02763579 (IMpower133)	A: Chemotherapy +atezolizumab; Control: Chemotherapy + placebo	4.5	A:58.1; Control: 57.6	Chemotherapy +atezolizumab:10.5; Chemotherapy + placebo:2.5	34(At 18 months)	2
NCT03043872 (CASPIAN)	A: durvalumab + tremelimumab+ Chemotherapy; B: durvalumab+ Chemotherapy; Control: Chemotherapy	A:6; B:8	A:55; B:46; Control:52	A:14; B:5; Control:<1	B:22.9(at 2 years)	B:2.4
NCT04063163 (ASTRUM‐005)	A: Serplulimab+ Chemotherapy; Control: placebo+ Chemotherapy	20.8	A: 33.2; Control: 27.6	A:9.5; Control:5.6	43.1(at 2 years)	4.5

The failure of the KEYNOTE‐604 and ECOG‐ACRIN EA5161 studies left us wondering whether PD‐1 monoclonal antibodies in combination with chemotherapy for ES‐SCLC could provide benefit, but the ASTRUM‐005 study has shown us again that similar study designs, drug mechanisms of action, and results may be different. It is necessary for us to continue the later studies to obtain more positive results. However, some scholars believed that the limitations of the study led to the exact action of Serplulimab still needing to be refined.^42^ The ASTRUM‐005 study population was contentious and differs from SCLC patients in the United States. The ASTRUM‐005 research comprised approximately two‐thirds of the Asian population and a higher number of nonsmokers than other clinical studies, limiting direct comparisons between trials.^42^ The researcher speculated that the recruited individuals may have other tissue types of lung cancer that were more responsive to immunotherapy, which could affect the study results.^42^ To treat SCLC, there were no randomized controlled trials that directly contrast the effectiveness of PD‐1 inhibitors and PD‐L1 inhibitors.^42^

The use of ICIs in SCLC for first‐line treatment is still confined to combination chemotherapy. New combination modalities of ICIs are being investigated to improve the benefit of ICIs in the treatment of SCLC. Currently, the combination of ICIs and radiotherapy is also being explored, and the clinical trial (NCT04562337), which used Adebrelimab in combination with chemotherapy sequential radiotherapy for the first‐line treatment of ES‐SCLC, has shown initial effectiveness, and a phase II study of Durvalumab combined with Ablative Radiation for ES‐SCLC is underway.

### 1.3 Second‐line treatment

The IFCT‐1603 trial included objective response rate(ORR) as the primary endpoint. And 73 patients were assigned at random to receive Atezolizumab or chemotherapy in a 2:1 ratio. According to the study's findings, the 6‐week ORR in the A arm was 2.3% and 10% in the B arm. The mPFS was 4.3 and 1.4 months, respectively. OS did not differ significantly between the A and B arms, which were 9.5 and 8.7 months, respectively (HR = 0.84, *p* = 0.6).^43^

The CheckMate 331 clinical trial evaluated the effectiveness and safety of Nivolumab with chemotherapy for the second‐line treatment of SCLC, with OS as the primary endpoint. And 569 individuals with ES‐SCLC or LS‐SCLC and an ECOG status of 0 or 1 were assigned at random to the Nivolumab or chemotherapy groups in a 1:1 ratio. OS in the A arm did not significantly improve, with mOS of 7.5 months and 8.4 months in the two arms (HR = 0.86, *p* = 0.11), 6‐month OS rates of 54.5% and 59.9%, and 12‐month OS rates of 36.6% and 34.1%, respectively, and mPFS of only 1.4 months in the A arm compared to 3.8 months in the Control arm (HR = 1.41). In the A arm compared to the Control arm, the median duration of response (mDOR) was better (8.3 vs. 4.5 months), and the safety profile was superior^44^ (Table 3).

**TABLE 3:** Second‐line, Third‐line, and maintenance treatment for SCLC

Second‐line treatment								
NCT ID	Stage	Number	Treatment		Endpoint	Progression‐Free‐Survival (month)	Overall survival (month)	
NCT03059667 (IFCT‐1603)	II	73	A:atezolizumab; Control: topotecan or re‐induction of initial chemotherapy for up to 6C		6 weeks ORR	A:1.4; Control:4.3; HR2.26, P = 0.004	A:9.5; Control: 8.7; HR 0.86, *p* = 0.11	
NCT02481830 (CheckMate 331)	III	569	A: Nivolumab; Control: chemotherapy with either topotecan or amrubicin		OS	A:1.4; Control:3.8; HR 1.41, P not tested	A:7.5; Control: 8.4; HR 0.70, *p* = 0.007	

Third‐line treatment								
NCT ID	Stage	Number	Treatment		Endpoint	ORR (Percent)	PFS (month)	OS (month)
NCT02628067 (KEYNOTE‐158)	II	107	A: Pembrolizumab		ORR	18.7	2.0	8.7
NCT02054806 (KEYNOTE‐028)	Ib	SCLC(24)	A: Pembrolizumab		ORR	33.3	1.9	9.7
NCT01928394 (CheckMate 032)	I/II	randomized cohort(243)	A:nivolumab1mg/kg + ipilimumab 3 mg/kg B: nivolumab3mg/kg + ipilimumab 1 mg/kg Control: nivolumab 3 mg/kg		ORR	A:21.9; Control:11.6	A:1.5; Control:1.4	A:4.7; Control: 5.7

Maintenance treatment								
NCT ID	Stage	Number	Treatment	Endpoint	PFS (month)		OS (month)	
NCT02538666 (Check‐Mate 451)	III	834	A: nivolumab + Ipilimumab; B: nivolumab; Control: placebo	OS	A:1.7 B: 1.9 Control:1.4; A VS control: HR 0.72 B VS control: HR 0.67		A:9.2 B:10.4 Control:9.6; A VS Control: HR 0.92; *p* = 0.3893; B VS Control: HR 0.84 (*p* not tested)	

### 1.4 Third‐line treatment

Based on KEYNOTE‐158 (Group G) and KEYNOTE‐028 (Group C1), the indication for Pembrolizumab for the treatment of SCLC received accelerated approval from the FDA in June 2019. A‐ arm had ORR of 18.7%, mOS of 9.1 months, and mPFS of 2.0 months, according to the KEYNOTE‐158 study,^45^ which was similar to the outcomes of the NCT02054806 trial,^46^, ^47^ a non‐randomized, multi‐cohort phase Ib “basket” study of 450 PD‐L1‐positive patients with more than 20 solid tumors. The SCLC cohort included 24 patients with advanced disease who had been treated and were intolerant to standard therapy. The trial reported a median ORR(mORR) of 33.3 percent, DOR of 19.4 months.

CheckMate 032 was a trial that could be included in this group regardless of PD‐L1 expression. In 2016, the ORR, which was the main study endpoint, was 10%, 23%, and 19% in the Control, A, and B arms, respectively^48^ (Table 3). The mPFS was 1.4, 2.6, and 1.4 months, respectively. The control arm and A‐arm had 1‐year OS rates of 33% and 43%, respectively. In light of the findings of the 2018 report, the 109 patients who received third‐line Nivolumab alone had a median follow‐up of 28.3 months, an ORR of 11.9 percent, and mDOR of 17.9 months. The mOS was 5.6 months, and the mPFS was 1.4 months.^49^ The OS rates were 28.3% at 12 months and 20.0% at 18 months, respectively. And 243 patients in the CheckMate 032 randomized cohort study who had progressed after receiving chemotherapy,^50^ with ORR as the primary endpoint. The ORRs for the Control arm and A‐arm were 11.6% and 21.9%, respectively. The 2‐year survival rates were 17% and 30%, respectively, with mDORs of 15.8 and 10.0 months. The mPFS was 1.4 and 1.5 months, respectively, with PFS rates of 15.9% and 9.5% at 6 and 12 months in the Nivolumab monotherapy group and 22.1% and 11.9% in the A‐arm. The mOS for the A arm was 4.7 months, compared to 5.7 months for the Control arm. The A arm improved the primary endpoint (ORR) significantly more than the Control arm, but the combination increased toxicity, with higher rates of grade 3–4 treatment‐related adverse events (TRAEs) in the A‐arm (37.5% vs. 12.9%)^50^, ^51^ (Table 3).

### 1.5 Maintenance treatment

CheckMate 451 used OS as the primary endpoint of the clinical trial. The goal of this clinical trial was to assess Nivolumab + Ipilimumab as maintenance therapy after first‐line chemotherapy in patients with ES‐SCLC. In a 1:1:1 ratio, 834 patients were randomly assigned to one of three groups: placebo, nivolumab alone, or Nivolumab plus Ipilimumab. The mOS in the A arm was 9.2 months, 10.4 months in the B arm, and 9.6 months in the Control arm. The primary endpoint of OS was not met, and the OS rates in the three groups were comparable^52^ (Table 3).

### 1.6 Summary

Compared with chemotherapy alone, the improvement of OS by ICIs combination chemotherapy is still limited. Although ICIs evolved rapidly, only a small proportion of SCLC patients can benefit from ICIs, and there has been no significant progress in second‐line, maintenance, and LS‐SCLC treatment. The exploration of resistance mechanisms of ICIs as well as biomarkers remains challenging. Additional targets and combination modalities to enhance the benefit of ICIs in SCLC treatment were being investigated. We anticipate that shortly, as we further investigate SCLC, new targets and new ICIs combination treatment strategies will bring longer survival benefits to more SCLC patients.

## ACKNOWLEDGMENT

The National Administration of Traditional Chinese Medicine: The Seventh Batch of National Traditional Chinese Medicine Experts Academic Experience Inheritance Project, the National Natural Science Foundation of China (No. 81403220), the Tianjin Health and Family Planning‐High Level Talent Selection and Training Project, and the National key research and development (R&D) plan(2018YFC1707400) have all contributed to this work.

## AUTHOR CONTRIBUTIONS

**Longhui Li:** Writing – original draft (equal). **Yangyueying Liang:** Writing – original draft (equal). **Minghui Yu:** Writing – original draft (equal). **Lu Zhao:** Writing – original draft (equal). **Qingyun Mei:** Writing – original draft (equal). **Yongchao Yu:** Writing – original draft (equal). **Na Wang:** Writing – original draft (equal). **Dou Zhang:** Writing – original draft (equal). **Ziwei Wang:** Writing – original draft (equal). **Yingjie Jia:** Writing – original draft (equal). **fanming Kong:** Writing – original draft (equal).

## CONFLICT OF INTEREST

This article contains no conflicts of interest.

## Data Availability

Data sharing is not applicable to this article as no new data were created or analyzed in this study.
